# The Relationship between the Glenohumeral Joint Internal Rotation Deficit and the Trunk Compensation Movement in Baseball Pitchers

**DOI:** 10.3390/medicina57030243

**Published:** 2021-03-05

**Authors:** Shih-Chung Cheng, Ting-Yu Wan, Chun-Hao Chang

**Affiliations:** 1 Graduate Institute of Athletics and Coaching Science, National Taiwan Sport University, Taoyuan 333325, Taiwan; iampowerguard@gmail.com; 2 Graduate Institute of Sports Science, National Taiwan Sport University, Taoyuan 333325, Taiwan; hao781106@gmail.com

**Keywords:** baseball, glenohumeral joint internal rotation deficit, pitching, kinematics, biomechanics

## Abstract

Background and objectives: Glenohumeral joint internal rotation deficit (GIRD) is commonly observed in the dominant arm of baseball pitchers and is limited by horizontal adduction motions. We inferred that when pitchers’ generation of internal shoulder rotation and horizontal adduction activity is limited, they may generate compensation movements in other body parts. This study aims to investigate whether pitchers with GIRD generates trunk compensation during pitching where pitching targets were on the lower corner of their non-dominant side. Design: Case-control study. Setting: Elite senior high school baseball. Participants: Twenty-five senior high school baseball pitchers participated in this study. Twelve pitchers with GIRD were assigned to the experiment group, and the remaining 13 participants to the control group. Main outcome measures: Glenohumeral internal/external rotation of both arms and internal/external rotation of the bilateral hip joints were measured. The kinematic values of the trunk when pitching to a target were measured using high-speed infrared cameras. Results: Pitchers with GIRD exhibited significantly greater upper trunk rotation toward the non-dominant side when a baseball was released from their hand (27.39 ± 6.62 degrees), compared with non-GIRD pitchers (20.42 ± 5.97 degrees) (*p* < 0.05). The total rotation of the pivot leg of pitchers with GIRD (67.54 ± 7.84 degrees) was significantly smaller than that of pitchers without GIRD (74.00 ± 7.07 degrees) (*p* < 0.05). Conclusions: GIRD in the dominant arm affected upper trunk rotation during pitching and was associated with the hip range of motion. Future studies could conduct a longitudinal study regarding the relationship between GIRD and other joint injuries of the lower limbs.

## 1. Introduction

Pitching is a complex systematic motion of the kinetic chain, starting from the lower limbs, in which the force is transmitted to the body trunk and the scapula before being transmitted to the upper distal joints [1,2]. Matsuo et al. (2001) and Werner et al. (2008) suggested that in the late cocking phase of throwing a ball, greater external joint rotation can increase the ball velocity [3,4]. However, the pulling force form the tendons to the growth plate on the humeral neck could lead to osseous adaptation and subsequent soft tissue adaptation, which results in a decrease in glenohumeral internal rotation of the dominant arm for more than 20 degrees comparing with the non-dominant arm. The phenomenon is called glenohumeral internal rotation deficit (GIRD). The adaptations also lead to a deficit in the total rotation of the dominant arm and a deficit in horizontal adduction rotation [5,6]. GIRD has been found to affect shoulder-muscle strength and scapula control and can easily cause injuries in upper limbs, such as shoulder impingement, glenoid labrum damage, ulnar collateral ligament injury [7,8,9,10]; moreover, GIRD affects the transmission of the overall kinetic chain. However, studies on GIRD in sports have mostly focused on the shoulder and elbow joints and have rarely examined the body trunk and lower-limb joints.

Chen (2012) found that when young pitchers (aged under 16 years old) with GIRD made fast and straight pitches, most balls could not hit the target but drifted towards the dominant side (i.e., a ball thrown by a right-handed pitcher would drift to the right side) [11]. However, this phenomenon was not significant in pitchers in senior high school (aged 16–18 years old) [11]. Cheng (2021) investigated in the kinematic characters of the knee in the leading leg and found that pitchers with GIRD had greater counter movement in the knee joint than pitchers without GIRD. In Cheng’s study, GIRD is implied to be a potential risk factor causing anterior cruciate ligament injury for baseball pitchers [12]. However, the reason causing the counter movement in the knee joint was not explained.

We suspected that baseball pitchers with GIRD might tend to use trunk rotation as compensation to adjust ball placement and subsequently cause counter movement at the knee of the leading leg.

Understanding the trunk compensatory methods of pitchers with GIRD and deficit in hip joint activities can elucidate changes in the kinetic chain of their trunk and lower limbs. The purpose of this study was to understand whether trunk compensation movement was produced in the pitching process of pitchers with GIRD. Additionally, the measurement of bilateral hip joint rotation could elucidate whether pitchers with GIRD have special adaptations for hip joint activities.

## 2. Materials and Methods

### 2.1. Subjects

Twenty-seven male participants were recruited in this study. The inclusion criteria were (1) baseball pitchers in the senior high school and (2) use overhead pitching skill. They were separated into two groups, including experiment group and control group. Twelve pitchers with GIRD were enrolled into the experiment group, and thirteen pitchers without GIRD were enrolled into the control group. All participants were right hand dominant, and all of them used overhead pitching skill. The exclusion criteria were (1) history of the shoulder surgery and (2) history of shoulder, trunk, lumbar, hip injuries within 6 months. All participants gave their written consent to take part in this study and allow disclosure of their anonymized personal details. This study has been granted ethical approval by the University Institutional Review Board on 5 May 2015 (FJU-IRB F-034) and was completed in 2018.

### 2.2. Procedures and Instrumentation

A goniometer (OSSUR, Iceland) was used for measuring joint range of motion in this study. We used data obtained from shoulder ROM to separate participants into the experiment and the control groups. 

**Figure 1:**
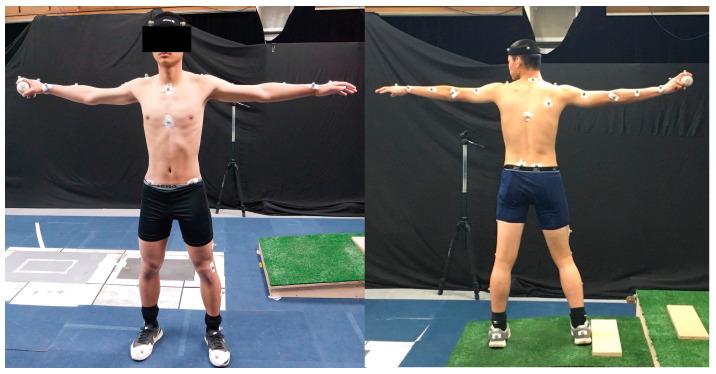
Reflective markers attached to the front and back of a participant.

Participants were lying in the supine position when their GHJ rotation was measured [5,13]. Participants were lying in the prone position when their hip joint rotation was measured. Next, we measured pitching movements using a motion capture system (VICON T40, Oxford Metrics Ltd., Oxford, UK) shooting with 10 infrared cameras at a frequency of 200 Hz. Before the measurement of indoor pitching movements, we attached 40 balls with reflective markers to participants’ bodies with the Plug-In-Gait model (Figure 1).

**Figure 2:**
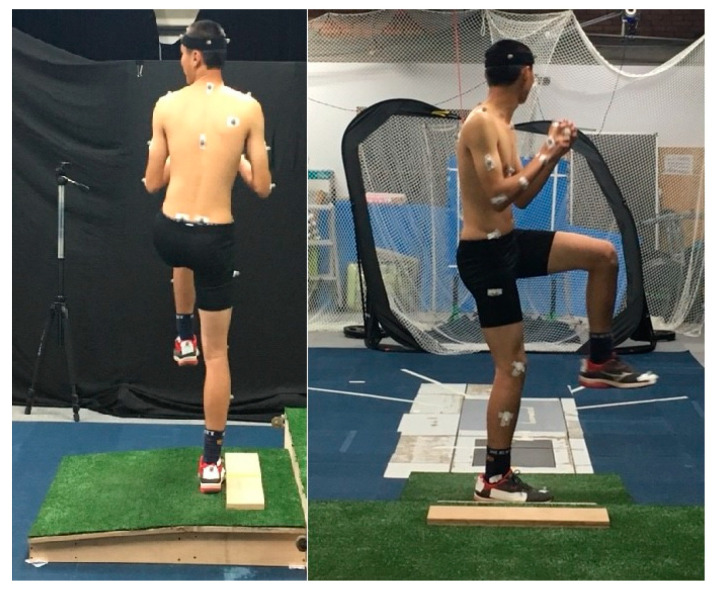
Side view and back view of a participant during the pitching test.

The pitching was performed on a self-made 5-inch indoor pitcher’s mound in the laboratory. A force plate (Kistler type 9287A, Winterthur, Switzerland) with a frequency of 1000 Hz was placed in front of the mound. The pitching target was a rectangular object with a height of 20 cm and a width of 15 cm, which was placed 5.85 m away from the pitcher’s mound. A total of 25 cm to the left of the center of the pitcher’s mound was set as the bottom left corner position of the strike zone. Ground clearance was 100 cm and calculated based on the ratio of the pitch distance in the laboratory (5.85 m) and the actual length of a baseball field (18.44 m). All participants had to place their pivot leg in a set position or stretch position on a pitching rubber, aligning it to the rubber’s centerline (Figure 2).

The moment that the participants’ leading leg stepped on the force plate was recorded as the ground-contact time of their leading leg, and the ground-contact time was calculated from when they started pitching. The baseball release time started at the moment when the distance increased between the distal interphalangeal joints of the index finger and the center of two reflective markers attached to the baseball, and time calculation stopped when participants’ pitching was completed. During this process, participants were encouraged to pitch with full strength, and data were collected from 5 successful pitches into the target.

### 2.3. Statistical Analysis

We used SPSS for Windows 20.0 to conduct statistical analysis; all values are shown with mean ± standard deviation. Descriptive statistics were used to analyze all participants’ demographic information; an independent-sample t-test was used to compare differences in the range of motion for the GHJs, trunk kinematic values, and hip joint rotation between the experimental (GIRD) group and control (non-GIRD) group. A matched-sample t-test was used to compare differences in the range of motion for bilateral GHJs and hip joints between the groups. The significance level was set to α = 0.05, and Cohen’s d was used to calculate the effect size of the trunk kinematic results. To have an 80% probability of detecting a significant difference in trunk rotation movement, we needed to enroll 24 participants, assuming an overall standard deviation in trunk rotation and a 2-tailed alpha-level of 5%.

## 3. Results

Twelves pitchers with GIRD and thirteen pitchers without GIRD participated in this study (Table 1). Table 2 shows the range of motion values of GHJ rotation for the GIRD and non-GIRD groups. Internal rotation of the dominant arm of the GIRD group was significantly smaller than that of the non-GIRD group (*p* = 0.009). In terms of external rotation, the GIRD group did not exhibit a corresponding increase in the external rotation due to reduced internal rotation of the dominant arm and was significantly smaller than that of the non-GIRD group (*p* = 0.025). Furthermore, the internal range of motion for the non-dominant arm of the GIRD group was significantly greater than that of the non-GIRD group (*p* = 0.042).

Table 3 and Table 4 present the kinematic results of the upper trunk and pelvis of the GIRD and non-GIRD groups. The results suggested that the GIRD group exhibited larger rotations when releasing the balls compared with the non-GIRD group (*p* = 0.011). Additionally, from the leading leg’s contact with the ground to the release of the ball from the pitcher’s hand, the total upper trunk rotation of the GIRD group tended to be greater than that of the non-GIRD group (*p* = 0.079), and the effect size was above 0.7. Furthermore, the overall pitching time of participants in the GIRD group was significantly longer than that of the non-GIRD group. In terms of ball velocity, no significant differences existed between the groups, but the non-GIRD group had a tendency to be faster than the GIRD group (*p* = 0.067), and the effect size was also greater than 0.7.

Table 5 presents the results of the internal and external rotation of the hip joints of the GIRD group and the non-GIRD group. The total pivot leg rotation of the GIRD group was significantly smaller than that of the non-GIRD group. The mean difference in pivot leg internal rotation of the two groups was 5°; even though such a difference was not statistically significant, the GIRD group’s hip joints tended to have a smaller pivot leg internal rotation (*p* = 0.06).

## 4. Discussion

This study aimed to observe the upper trunk rotation degree of senior high school baseball pitchers during pitching. The results suggested that the upper trunk rotation angle of the GIRD group during ball release was significantly larger than that of the non-GIRD group, which was consistent with our hypotheses. This indicated that pitchers with GIRD would increase compensation movements in their upper trunk toward their non-dominant side when rotating their trunk in the direction of the home base. This finding is consistent with the proposition of Cheng et al. (2011) [14]. Specifically, these researchers proposed that pitchers with GIRD who are older than senior high school age may maintain their baseball control ability through increased upper trunk compensation movements due to limitations of the GHJs in their dominant arm. Additionally, we integrated our results with the findings of Cheng et al. (2020) and found that senior high school pitchers with GIRD would increase the degree of external rotation of the leading leg femur relative to the tibia when releasing baseballs from their hand [12]. The trunk is adjacent to the pelvis, and increasing the upper trunk’s rotation toward the non-dominant side would produce a force of external rotation in the femur, which is connected to the pelvis. The tibia can be assumed to be fixed on the ground when the leading leg contacts the ground. Therefore, the femur would increase movements relative to the external rotation of the tibia in the dynamic process of pitching. 

We compared the upper trunk rotation degrees of pitchers in the non-GIRD group (20.42 ± 5.97°) in our study with their counterpart group (20 ± 5°) in the study by Chou et al. (2018). The values of the two groups were similar, indicating that no excessive rotation changes were generated during ball release because the strike zone was set as the pitching target. However, GIRD group pitchers in the two studies exhibited significantly different upper trunk rotation degrees (27.39 ± 6.62° in our study and 7 ± 10° in that of Chou et al. (2018) [15]. Overall, the results of this study and Chou et al. show that pitchers with GIRD may generate compensation movements through different parts of their bodies when the pitching target is in different locations. Relevant studies have found that such pitching movements with relatively large changes influence pitching performance [16,17]. However, few studies have investigated the consistency of pitching pose and the risk of sports injuries; they have merely hypothesized that reduced pitching pose consistency may interfere with pitching flow and increase compensatory movements [16]. The question of whether pitchers’ repeated and accumulated changes in pitching style affect their overall pitching performance and risk of being injured requires further research.

In our study, even though the GIRD group exhibited significant increases in upper trunk rotation, their ball velocity was not faster, indicating that their force was lost in the transmission process. They failed to pitch with optimally coordinated trunk rotation. This can affect the overall pitching movement and generate a greater burden on upper limb joints. Studies have suggested that abnormal trunk rotation coordination can increase pressure on the shoulder-elbow joints [16,17]. Therefore, it can be inferred that adopting inappropriate pitching strategies through trunk rotation might not improve pitchers’ pitching performance and could increase their risk of sports injuries.

Our results regarding the internal and external rotations of GHJs of the dominant arm of all participants in this study were consistent with rotation characteristics found in relevant studies [18,19,20]. Specifically, participants exhibited increased external rotation and decreased internal rotation in their dominant arm compared with their non-dominant arm. The data of the GIRD group indicated that the average internal rotation deficit of the dominant arm was 25.04 ± 5.02°. These values are similar to the internal and external rotation values (26 ± 6°) of Taiwanese senior high school pitchers with GIRD measured by Chou et al. (2018) [15]. The values of such internal rotation deficit also exceeded the threshold value (20 to 25°) of movements, which is prone to causing injuries in upper limb joints, as suggested by relevant studies [7,9,21]. Our participants with an internal rotation deficit greater than 20° had a total rotation angle deficit of more than 5° simultaneously (14.09 ± 6.06°). The values were similar to the total rotation angle deficit (~13°) of the same ethnic group measured by Chou et al. (2018) [15]. This suggested that with the excessive reduction in internal rotation of the dominant arm, pitchers with GIRD did not generate a sufficient corresponding increase in their external rotation. Wilk et al. (2011) noted that if the total rotation deficit exceeds this angle range, then the risk of upper limb injury would increase [22]. Our results also reveal that considerable differences can exist in the range of GHJ motion among healthy participants of the same ethnic group. The results and data of trunk and hip joint measurements indicated differences between the GIRD group and the non-GIRD group.

Additionally, we found that the internal rotation of the GIRD-group participants’ non-dominant arm was significantly larger than that of non-GIRD participants. Such a finding was unexpected and is inconsistent with the measurement results in the previous studies by Chou et al. (2018) and Nakamizo et al. (2008), but consistent with the results of the study by Noonan et al. (2015) [15,23,24]. The humerus of the non-dominant arm withstands less pressure from pitching and does not generate adaptive backward tilting of the humeral head in response to larger external rotation compared with the dominant arm. Therefore, GHJ rotation of the non-dominant arm can predict the tilting-backward degree of the congenital bilateral head of the humerus. Our results suggested greater internal rotation of the non-dominant arm of the GIRD group, and it could be inferred that this group had less humeral head backward tilting. Previous studies have also proposed that smaller humeral head backward tilting of the non-dominant arm can increase pitchers’ risk of injury [25,26]. We summarized the aforementioned results of internal rotation of the non-dominant arm of the GIRD group. Overall, few studies have been able to verify the reasons for such a phenomenon, but it may be related to the direction of congenital humeral head rotation and sports injuries. Future research can continue to observe this phenomenon.

## 5. Conclusions

Pitchers with GIRD exhibited significantly greater upper trunk rotation during ball release compared with non-GIRD pitchers. This finding was consistent with our research hypotheses and suggested that pitchers with GIRD indeed use large trunk rotations to compensate for their ball release. In addition to trunk movements, we found that the total rotation of the hip joints of the pivot leg in the GIRD group was significantly smaller than that of the other group. The limitation of the range of hip joint motion is similar to that of the sacroiliac joint and the lumbar spine. Specifically, we gained knowledge that pitching is a bottom-up transmission movement of the overall kinetic chain. The aforementioned results contribute to the understanding of the relationship between the range of GHJ motion and upper trunk and hip joint motion.

## Figures and Tables

**Table 1:** Comparison of participants’ demographic information between the two groups.

	GIRD Group	Non-GIRD Group	*p* Value
Age (years)	16.64 ± 0.95	17.08 ± 1.23	0.339
Height (cm)	178.92 ± 3.82	179.15 ± 5.68	0.904
Weight (kg)	76.92 ± 6.6	76.59 ± 11.1	0.931
Weekly baseball practice time (hours)	21.08 ± 2.02	22.31 ± 1.7	0.114
Pitching experience (years)	5.08 ± 1.98	5.15 ± 2.18	0.933
Total baseball experience (years)	6.67 ± 1.67	7.04 ± 1.42	0.554
Number of participants	12	13	

**Table 2:** Glenohumeral joint rotation among participants.

	GIRD Group	Non-GIRD Group	*p*
Right external rotation (degree)	96.42 ± 8.42	97.42 ± 6.6	0.741
Right internal rotation	40.71 ± 3.50	48.65 ± 9.01	0.009 *
Right horizontal adduction	14.13 ± 2.86	15.42 ± 3.05	0.285
Right total rotation	137.13 ± 7.16	146.08 ± 10.96	0.025 *
Left external rotation	85.67 ± 6.71	90.88 ± 7.11	0.072
Left internal rotation	65.75 ± 5.19	59.62 ± 8.52	0.042 *
Left horizontal adduction	18.38 ± 4.28	18.81 ± 2.95	0.770
Left total rotation	151.42 ± 8.52	150.5 ± 9.52	0.803

Note: * indicates a significant difference between the two groups (*p* < 0.05).

**Table 3:** Kinematic analysis of participants’ upper trunk.

Upper Trunk Motion (Degree)	GIRD Group	Non-GIRD Group	*p* Value	Cohen’s *d*
Upper trunk rotation (SFC **)	−90.06 ± 9.64	−88.32 ± 11.84	0.692	0.1605
Upper trunk rotation (REL ***)	27.39 ± 6.62	20.42 ± 5.97	0.011 *	1.1082
Upper trunk total rotation	117.45 ± 11.55	109.12 ± 11.26	0.079	0.7307
Upper trunk roll (SFC)	−4.50 ± 8.01	−4.33 ± 5.13	0.950	0.0255
Upper trunk roll (REL)	24.70 ± 6.52	23.74 ± 6.21	0.710	0.1509
Upper trunk flexion (SFC)	−1.94 ± 10.06	2.99 ± 7.45	0.175	0.5605
Upper trunk flexion (REL)	29.44 ± 4.87	33.47 ± 6.96	0.109	0.666

Note: * indicates a significant difference between the two groups (*p* < 0.05). ** SFC: stride foot contact. *** REL: ball releasing.

**Table 4:** Kinematic analysis results of participants’ pelvis and ball velocity.

Pelvic Rotation (Degree)	GIRD Group	Non-GIRD Group	*p* Value	Cohen’s *d*
Pelvic rotation (SFC **)	−63.63 ± 10.85	−60.03 ± 11.35	0.427	0.3239
Pelvic rotation (REL ***)	17.25 ± 6.15	13.31 ± 10.91	0.283	0.44
Total pelvic rotation	80.88 ± 11.34	73.91 ± 11.88	0.148	0.5239
Ball velocity (km/hr)	113.21 ± 9.52	119.79 ± 7.52	0.067	0.7709
overall pitching time(s)	0.195 ± 0.024	0.174 ± 0.018	0.024 *	0.996

Note: * indicates a significant difference between the two groups (*p* < 0.05). ** SFC: stride foot contact. *** REL: ball releasing.

**Table 5:** Hip joint rotation of participants in the two groups.

Rotation Direction (Degree)	GIRD Group	Non-GIRD Group	*p* Value
Pivot leg external rotation	37.75 ± 7.97	38.38 ± 4.90	0.811
Pivot leg internal rotation	29.79 ± 6.77	35.62 ± 7.86	0.06
Pivot leg total rotation	67.54 ± 7.84	74.00 ± 7.07	0.041 *
Leading leg external rotation	36.21 ± 7.45	38.42 ± 5.57	0.406
Leading leg internal rotation	29.75 ± 7.28	32.31 ± 7.20	0.387
Leading leg total rotation	65.96 ± 6.53	70.73 ± 7.65	0.109

Note: * indicates a significant difference between the two groups (*p* < 0.05).

## Data Availability

The data presented in this study are available on request from the corresponding author. The data are not publicly available due to agreement with subjects via the ethical consent form.
